# “Essential Not Optional”: Spiritual Care in Australia during a Pandemic

**DOI:** 10.1177/1542305020985071

**Published:** 2021-03-17

**Authors:** Heather Tan, Cheryl Holmes, Eleanor Flynn, Leila Karimi

**Affiliations:** Spiritual Health Association, Australia; University of Divinity, Australia; College of Science, Health and Engineering School of Psychology and Public Health, La Trobe University, Australia

**Keywords:** Spiritual care, COVID-19, Australia, pastoral care, chaplaincy

## Abstract

This paper focuses on the impact of COVID19 in Australia. Three areas were investigated: professionalism, contrasting hospital and aged care services and “business as usual”? Impact was low overall, the timing being pre-second wave impact. Two areas of weakness were highlighted: depleted spiritual care teams due to standing down non-professional staff and uncertainty about the role of Chaplains in the care of other staff. Further study of second wave impact is recommended.

## Introduction

In Australia, as in many locations world-wide, the need to commit to the provision of spiritual care as an integral part of the healthcare system, has been a focus for some time (Ferrell et al., 2020; Puchalski et al., 2014). Although the spreading of the COVID19 virus across the continents was delayed in Australia compared to Europe and North America, the chance to take part in this world-wide survey, investigating the impact of this virus on spiritual care practice, was a very important opportunity.^1^

There has been some early investigation into the impact of COVID19 on the provision of spiritual care in the health systems within Australia such as (Drummond & Carey, 2020) which presents an aged care case study. This paper offers a broader perspective on the impact in Australia during the first half of 2020.

In this paper the areas of professionalism within the spiritual care sector of health care, the offering of spiritual care in the aged care sector compared to hospitals and the degree to which the impact of COVID19 could be seen as an opportunity to bring permanent change to the provision of spiritual care. It should be noted that the terms spiritual care, pastoral care and chaplaincy are used somewhat interchangeably in the Australian setting.

## Methods

Data contributed by Australian responders were analysed using qualitative and quantitative data analysis procedures. Additional qualitative data collected from Australian spiritual care managers at health care venues was also analysed.

Thematic analysis, utilising categories and themes was undertaken by three of the authors in exploring the qualitative data. Three themes emerged: the extent to which spiritual care practitioners/chaplains were regarded as professionals in the health care system; differences in the responses of Aged Care Services compared to main-stream hospitals and the degree to which the impact of COVID19 resulted in a change of practice rather than business as usual. This latter theme also gave an indication of how spiritual care practitioners found the overall experience.

Descriptive analysis (such as frequency, mean) and non-parametric analysis of Chi square were used to analyse the quantitative data and Fisher exact test for contingency tables were reported when sample sizes were small. SPSS (v. 26) was used for data analysis.

## Results

### Quantitative Data

#### Professionalism

Demographic and work characteristics of the participants are presented in Table 1. The average age of spiritual care practitioners was 58 years, the majority being female (63%). The level of qualifications of participants was merged into two categories demonstrating almost equal distribution between undergraduate (*n* = 93, 53%) and postgraduate levels (*n* = 81, 47%). Similarly, with membership of a professional association, with 51% (*n* = 96) holding membership compared with 49% (*n* = 92) not. The majority of participants had regular supervision, intervision or other form of group reflection often or very often (*n* = 14,374%).

**Table 1.** Demographic and work characteristics of the participants (*n* = 199^a^).

	Frequency	Percent
Qualification
Undergrad	93	53.4
Postgrad	81	46.6
Membership of association
Yes	96	51.1
No	92	48.9
Supervision/intervision
No/rarely/sometimes	49	25.5
Often/Very often	143	74.5
Place of work
Hospital	70	44.6
Aged Care	66	42.0
Other	21	13.4
Access to COVID19 patient
Yes	32	22.1
No	113	77.9
Gender
Male	70	35.4
Female	124	62.6
Other	4	2.0
Religion/Faith
Christian Protestant	105	54.7
Christian Catholic	63	32.8
Humanist	3	1.6
Hindu	2	1.0
Buddhist	3	1.6
Others	16	8.3
State
NSW	53	26.2
NT	1	.5
QLD	13	6.4
SA	12	5.9
TAs	6	3.0
VIC	76	37.6
WA	23	11.4
Age (mean, SD)	57.84 (9.07)	
Years of experience (mean, SD)	9.61 (7.84)	

^a^*n* varies between 139 and 201 due to some missing data.

Non-parametric chi square tests were conducted between the three areas of qualifications, association membership and supervision (taking these as indications of their professional status within their institution), and participants access to COVID19 patients and provision of staff support. The statistical significance threshold was set at *p* < 0.05. Those who were members of a professional association had significantly (χ^2^ = 3.88, *P* < 0.05) higher access to COVID19 patients compared to non-members (67% vs 33%).

No significant differences were found between the level of qualification and access to COVID19 patients (χ^2^ = 0.035, *P* > 0.05). Those who were members of a professional association and had postgraduate qualifications demonstrated higher access to COVID19 patients (82%), however this was not statistically significant (χ^2^ = 3.00, *P* > 0.05). Those with more regular levels of supervision working at hospitals (as compared to aged care) had more access to COVID19 patients (74%) but again the results were not statistically significant (χ^2^ = 1.91, *P* > 0.05).

There was no significant difference between level of education and provision of staff support (χ^2^ = 2.55, *P* > 0.05). Those who were members of an association and had regular supervision provided higher levels of staff support (82%) and this was statistically significant (χ^2^ = 3.28, *P* < 0.05).

#### Hospital versus Aged Care Provision of Services

By grouping the participants’ responses by their place of work we saw that 42% of Australian participants worked in Aged Care services compared to 44.6% in hospitals and 13.4% in other places, including palliative care, mental health and prisons. Most reported that as the pandemic (at that time) had not affected Australia severely there had been no notable change of either practice or deemed need for spiritual care. Although access to COVID19 patients was much higher among those working in hospitals than aged care, 79% vs 21%, there were some Aged Care services with seriously ill residents (χ^2^ = 10.93, *P* < 0.05).

### Qualitative data

#### Professionalism

Free text data were analysed for the professionalism theme. While access to COVID19 patients was not referenced, there was a broader consideration given to whether spiritual care was considered ‘essential rather than optional’. For a number of services this was a time of being seen as ‘part of the team’.

*More integration with staff we are seen as part of the team. Spiritual care was named as essential to our service. (P1023 Female 54)*

For others the experience was of being dispensable or isolated.

*Most practitioners I know were either directed to work from home, or in their office on the phone. (P1300 Female 50)*

Data gathered from other sources at the time of the pandemic showed that across Australian hospitals the volunteer spiritual care workforce was stood down. This included visits by faith community representatives. For the hospital employed workforce there was greater interaction across disciplines, especially social work and psychology. Many acknowledged the need to promote the long-term values of a professional spiritual care work force with more adequate education and training.

#### Hospital versus Aged Care Provision of Services

The qualitative data analysis of aged care versus hospital showed that most of the responses from this sector were more positive than those from hospital-based workers;

*Several people who were dismissive of pastoral care came to see the value of the team during the pandemic*. (*P1546, Male 61)*

The few negative ones related to poor funding for spiritual care in the sector, or limitations on access to patients/residents. Most hospital based spiritual care workers reported that they were able to provide care using technology, or in a limited manner but did not feel as valued as their colleagues in aged care

*I have felt underused and not included apart from regular email updates*. (*P651, Male 68).*

The positive responses related to how well their hospital was prepared or how much the hospital valued the chaplaincy team. Though one participant who worked across hospital and aged care reported that the whole team was stood down.

The number of practitioners who provided staff support in Australia was lower than in Europe and North America and is perhaps partially explained by this response;

*… the number of workplaces that have changed their policy so that spiritual care staff cannot give spiritual care to other staff. (P1018, Female 53)*

Business as Usual?

A number of issues were raised in response to the question: What was new/different during this time?

1. Lack of COVID19 patients:

*Australia was further behind Europe and USA. Because of this lag of approx. 1 month, we were able to prepare as we were watching the what was happening in Europe and USA. (P1053, Female 53)*

2. The use of technology: This included the use of telehealth and other technology by team members (many working from home) following up referrals with patients and family members.

*Telehealth and working from home both new. (P415, Male 68)*

3. Distancing rules: *My greatest new learning was the physical distancing with patients. I found this very difficult to not hold a hand or give a hug. …People dying by themselves was very hard to hear about*….(*P1018 Female 53)*
4. More time caring for other staff than usual:

*Tweeting daily a message for staff in the hospital. offering meditation for staff and debriefin*g. *(P309 Male 60)*

*We are much more recognised for our role in staff care than prior to the pandemic*. *(P1054 Male 66)*

5. Lack of specific faith community visits (non-paid staff not allowed on site)

*Our aged care Catholics and Anglicans missed the Priest and Deacon celebrating Mass once each month*. *(P464, Female 61)*

6.  The Overall Experience: In those sites with COVID19 patients there were quite a lot of changes, some dramatic, although at sites where spiritual care practitioners were regarded as high risk and not able to work, they may not have had much opportunity to experience this difference.

*Brought us into the 21st century - old dogs learning new ways of doing things. My electronic know how has increased enormously and I feel more confident to try things*. *(P1047 Female 59)*

## Discussion

### Professionalism

The results suggest that access to COVID19 patients and provision of staff support was higher if the spiritual care practitioners were members of a professional association and received more regular supervision. These, together with qualifications, could be seen as indicators of professional status (Swinton, 2013). It is impossible to draw any definitive conclusions from these results given the small number of COVID19 patients in Australian hospitals at the time of the survey and the limited numbers for comparisons. However, the need for an increased professional spiritual care workforce has already been identified in the Australian context (Eve & Phillips, 2019; Tan et al., 2020), and the need for a nationally consistent approach to spiritual care documented (Holmes, 2018). A recent study in Scotland found that credibility was identified as one of the benefits of professionalism (Snowden et al., 2020). It may be that those practitioners with a higher level of professionalism (i.e. post graduate qualifications, association membership and regular supervision), were perceived as more credible and therefore more fully integrated into the institution’s COVID19 response.

Together the quantitative and qualitative data paint a picture of the mixed models of spiritual care provision across participating sites. With all volunteers stood down the question of sustainability of a model based on a volunteer workforce must be raised. There is a need for a greater investment in a professional spiritual care workforce or the models will not move from a reliance on volunteers and chaplains paid by faith traditions. There is a need for further research to confirm whether professionally trained, supervised and certified practitioners make a difference to the quality of spiritual care for patients and staff and to the professional credibility of the practitioners within their institutions.

### Aged Care versus Hospital

Since only New South Wales and Victoria had more than a few cases at the time of the survey and the majority of responses are from those states, these are of more value in considering any learnings from the effect of the pandemic for Australian spiritual care services. Respondents from both hospitals and aged care predominantly said they were not allowed to visit COVID19 patients at all while a small minority could ‘visit’ using IPads from outside and even fewer could enter the patient’s room. Further work needs to be done to convince health service managers of the benefits of spiritual care for seriously ill people.

As indicated, those not paid by the health service were regarded as ‘volunteers’ and unable to visit most places in the pandemic. It was suggested that these people should be reclassified as contractors so they would be able to visit patients in the future. This change plus a better understanding of the benefits of spiritual care by hospital and aged care managers might allow both in house and volunteer spiritual care staff to access the appropriate personal protective equipment and visit patients who request or need spiritual care, until a more professional model can be widely utilised.

The structure and staffing of aged care services in Australia is currently the subject of a wide-ranging legal investigation, made more urgent by outbreaks of COVID19 in Victoria where 642 of the 672 deaths in Australia have occurred since the survey was undertaken. While the concept of spiritual care is discussed and some provided, there is not a specific funding stream for dedicated staff. Submissions to the Aged Care Royal Commission have suggested the benefits of moving spiritual care “from the margins to become an intrinsic part of the design of services” (Hampton, 2020).

### Business as Usual?

The data suggest that in many sites at the time of the survey it was largely business as usual. As has already been stated the two main reasons for this were: low COVID19 numbers at the time of the survey, and non-professional models of practice that excluded many spiritual care practitioners from on-site care of patients on grounds of their non-paid status within the health service or their age.

### Limitation

A significant limitation is that the timing of the survey came before a major second wave of infections in Australia, especially in the state of Victoria. Some of the data may well have been quite different had the survey been later. The qualitative data in this study lacks depth compared to the collected for focus groups or semi-structured interviews. One of the latter could well be considered in further studies in this area.

## Conclusion & Recommendations

This survey has highlighted potential problems in the provision of spiritual care in Australian health services, both in hospitals and aged care. Of particular note are:

1. The existence of multiple models of spiritual care provision, some of which include providers who do not meet the requirements of a professional spiritual care workforce, leading to them being easily stood down and consequently spiritual care teams seriously depleted when they are needed most.
2. Differing views and policies about the extent to which support of other staff is considered part of the spiritual care practitioners’ remit.

As times of crisis such as the COVID19 pandemic are known to highlight both strengths and weaknesses in systems, it is recommended that a further study be undertaken in Australia which covers the period of the second wave infection, hence potentially providing more conclusive data.

## Note

1 The article Snowden, A. (2021). Overall description of survey article. *Journal of Pastoral Care and Counselling*. (special Edition January 2021).
